# Assessing adaptive capacity in smallholder farming systems in Karonga, Malawi

**DOI:** 10.4102/jamba.v17i1.1644

**Published:** 2025-04-11

**Authors:** Chakufwa K. Munthali, Victor Kasulo, Mavuto Tembo

**Affiliations:** 1 African Centre of Excellence in Neglected and Underutilised Biodiversity, Faculty of Environmental Sciences, Mzuzu University, Mzuzu, Malawi

**Keywords:** adaptive capacity, biophysical components, socio-economic components, principal component analysis, Karonga district, Malawi

## Abstract

Climate variability is expected to have a negative impact on agricultural production, particularly in sub-Saharan Africa, including Malawi, where the agricultural sector is a crucial part of the economy. This study focusses on increasing our understanding on the ability of smallholder farmers in Karonga, Malawi, to adapt to climate variability. To achieve this, an integrated framework was used to assess the factors that influence the adaptive capacity of smallholder farming systems in Karonga. The integrated indicator-based framework was used to assess financial, social, human assets, knowledge and information, institutions and entitlements, flexible and forward-looking decision-making, gender and power dynamics, natural assets, physical assets and the frequency of floods. Data were collected through face-to-face interviews, focus group discussions and observations. A total of 38 indicators from literature and observations in the study area were entered and analysed in SPSS and Excel using principal component analysis. The survey results indicate that the aggregate adaptive capacity of respondents is low. Component indicators, such as ownership of livestock, support from the community, livelihood diversification and gender of the decision maker, as well as access to a mobile phone and inorganic fertiliser, and share of more productive land, increased the adaptive capacity of smallholder farmers. On the other hand, component indicators such as less productive land, deforestation, capital exclusion from food for work, age of household head, financial assets and gross annual income reduced the adaptive capacity of smallholder farmers to climate change.

**Contribution:**

The article presents an integrated framework that considers both biophysical and socio-economic factors for assessing adaptive capacity. This framework offers a better understanding of the adaptive capacity of farming systems at the smallholder farmer level. The study’s findings provide insights into the dynamic nature of adaptive capacity and identify factors that either enable or constrain adaptive capacity at various levels.

## Introduction

The consequences of climate change on communities and people’s livelihoods are severe. As global warming continues to exceed current levels, the effectiveness of adaptive strategies to minimise risk is greatly reduced. It is anticipated that crop output losses will escalate significantly beyond a global warming threshold of 2°C, regardless of any adaptation measures (Intergovernmental Panel on Climate Change [IPCC] 2022). Ecosystems are already experiencing the limits of adaptability. Beyond the limits of natural climatic variability, human-induced climate change has resulted in widespread detrimental effects, losses and damages to people and the environment, beyond natural climate variability.

The most vulnerable individuals and systems are seen to be disproportionately impacted across sectors and geographical areas. As natural and human systems are pushed beyond their capacity for adaptation, the rise in weather and climatic extremes has had some irreversible effects (IPCC 2022). The future of humanity is being threatened and challenged by the century-long global climate change and variability (Shikuku et al. 2017). Widespread degradation of Malawi’s ecosystem structure and natural adaptive capacity is occurring, and climate change has caused seasonal timing adjustments that have negative socioeconomic effects. Climate change, which includes an increase in the frequency and severity of extremes, has decreased food security and water security, making it more difficult to achieve the sustainable development goals; as a result, the area of adaptive capacity is becoming the field of more interest across the spectrum of social ecological and environmental change research. As a result, across the board in social-ecological and environmental change studies, adaptive capacity is gaining attention.

Climate change is defined as an alteration in the condition of the climate that lasts for a long time, usually decades or longer, and may be detected (e.g. by employing statistical tests) by changes in the mean and/or the variability of its properties, and climate variability is defined as deviations of climate variables at all spatial and temporal dimensions from a given mean state (including the occurrence of extremes, etc.) beyond the scope of individual weather events (IPCC 2022). Because of the inadequate adaptive capacity of the communities, sub-Saharan Africa (SSA) has been highlighted as being highly affected by climate change (Niang et al. 2014). Sub-Saharan Africa is particularly vulnerable to climate change-related hazards, such as increased temperature extremes, anomalies in precipitation, floods and droughts. Each year, these events put millions of people in danger; leave them maimed, homeless or food insecure and result in significant and expensive economic loss (IPCC 2022). There is rising worry that smallholder farmers in Sub-Saharan Africa (SSA), who mostly rely on rain-fed agriculture for their livelihood, may not be as well equipped to adjust to present and projected climatic changes as they should be.

The frequent occurrence of floods in Malawi is an indicator that the country is witnessing a rise in extreme weather, which has a big impact on the country’s population and economy (Mataya, Vincent & Dougill 2020). In 2016, 2.8 million people in Malawi were devastated by floods, while in 2023, tropical cyclone Freddy hit the country. Tropical Cyclone Freddy resulted in floods and mudslides, which devastated communities in southern Malawi, destroying houses, livelihoods and lives and saw at least 1076 fatalities. According to the government’s Department of Disaster Management Affairs (DoDMA), around 883 000 residences were impacted, causing 659 278 individuals to flee their homes and seek refuge in 747 displacement sites, including schools, churches, community centres and other locations. Karonga District was one of the most severely impacted areas by these two incidents, with an estimated total cost of damages and losses of more than $335 million (Government of Malawi 2016). Floods reduced smallholder farmers’ ability to adapt, which resulted in maladaptation (Mataya et al. 2020).

Depending on their environment, various people and societies will have varying capacities for adaptation. To prevent maladaptive consequences, it is critical to assess the elements that contribute to adaptive capacities in certain circumstances, as well as how those capacities vary even within the same community. According to Angeler et al. (2019:4), ecologists have defined adaptive capacity as ‘the latent potential of an ecosystem to alter resilience in response to change’. This definition directly refers to the characteristics of an ecosystem that support persistence during or after disturbance. Similar definitions are given by social scientists, who emphasise the biophysical and socioeconomic factors that support people’s capacity to foresee, adjust to and recover from the effects of environmental change on both an individual and community level (Sechindra et al. 2022).

Smallholders are those who manage areas ranging from less than one hectare to 10 hectares, such as fishermen, pastoralists, small-scale farmers and forest guardians. Family-oriented motivations, such as supporting the stability of the farm household system, utilising family labour primarily for production and reserving a portion of the yield for family consumption, are characteristics of smallholders (FAO 2017).

Because it is situated inside the Great Rift Valley plain, which is the most geologically active region when it comes to natural catastrophes, Malawi’s Karonga district is among the most susceptible to disasters linked to climate variability. Karonga District’s geological and environmental circumstances make it vulnerable to natural hazards such as earthquakes, droughts and floods (Manda 2017). Agricultural livelihoods in rural communities, which are mostly poor, are thought to be the most vulnerable to the effects of climate change (Singh, Deshpande & Basu 2017). Their rain-fed agriculture, which makes up most of the district’s economic activity GoM (2018a, 2018b, 2018c), is mostly dependent on a single, already-modified rainy season. A growing number of studies focussed mostly on farm-level adaptation techniques and strategies have been conducted in Malawi during the past 10 years (Phiri 2020). Few research studies have been conducted on the ability of smallholder farmers in Karonga to adapt to new climate patterns (Asfaw 2016). Finding out who among smallholder farmers are expected to be less able to adjust to climatic unpredictability allows organisations and government departments engaged in climate change adaptation programmes to find better ways to support smallholder farmers’ adaptation to climate variability in Karonga and Malawi in more efficient ways in their attempt to maintain their agricultural production. Over the course of the last 20 years, adaptive capacity has grown in importance as a factor in climatic variability, and research on the subject has expanded quickly (Montreux & Barnett 2017).

In addition to individual and social traits, biophysical or environmental elements within communities also have a role in determining the capacities required for humans to adjust to such manifestations of climate change. The definition of adaptive capacity used in this study is the collection of biophysical and socioeconomic factors that support people’s potential to foresee, adjust to and recover from the effects of environmental change (Engle 2011).

Development practitioners can better comprehend the elements that contribute to adaptive capability by using the helpful images that frameworks give. Actors can, however, make limited use of locally accepted evaluation frameworks to gain a deeper comprehension of the district’s smallholder farmers’ potential for adaptation. Furthermore, as the following highlights, the few frameworks that have been adopted concentrate on the socioeconomic factors that determine adaptive capacity while ignoring the biophysical factors. As a result, critical information in relation to understating the levels of adaptive capacity of smallholder farmers to climate variability is missed, which is key in the development of policy and interventions on climate change adaptation.

In this study, biophysical components of adaptation are defined as factors that are associated with the nature of the hazard and its first-order physical impacts, and a biological or social component associated with the properties of the affected system that act to amplify or reduce the damage resulting from these first-order impacts (Kapitza et al. 2021), and social economic components of adaptation are defined as factors that are associated with the sensitivity of a population to natural hazards and its ability to respond to and recover from their impacts, and they include community’s literacy level, employment status, income levels, housing ownership, age and sex distributions, religious beliefs, kinship levels and informal social support networks (Kapitza et al. 2021).

## Literature review

### Frameworks for understanding adaptive capacity of smallholder farmers to climate change and variability

The sustainable livelihoods framework (SLF) emphasises how human behaviour and the environment interact to shape livelihoods. The framework describes people’s potential in terms of their abilities, access to resources for a living and capacity to influence institutions. It also identifies the variables that limit or increase livelihood opportunities (Serrat 2017:21–26).

Many frameworks have strong links to the SLF and have adopted some of the SLF’s five ‘capitals’ (human, economic, social, physical and natural that encompass both socioeconomic and biophysical tenets of adaptive capacity), transformational structures and processes that encompass theories on livelihoods relating to institutions, organisations, policies and legislation that help to shape livelihoods as direct indicators of adaptive capacity at the community and household levels (UNDP 2017). This is an attempt to incorporate dynamic dimensions of adaptive capacity, as well as capital and resource-based components, into an analysis of adaptive capacity at the local level. However, most frameworks focus on human capital, economic capital and social capital leaving out physical and natural capital.

### The local adaptive capacity framework

To include resource-based elements as well as intangible and dynamic aspects of adaptive capacity in a local adaptive capacity analysis, the ‘local adaptive capacity framework’ (LAC) was created (Mesfin et al. 2020a). It begins by acknowledging that direct measurements of adaptive capability are not yet possible. Rather, LAC is predicated on an examination of the attributes that augment a system’s ability to adapt. With the underlying premise that positive impacts on these qualities should boost the system’s adaptive capacity, the framework lays forth five separates yet connected aspects of adaptive capacity.

**FIGURE 1:**
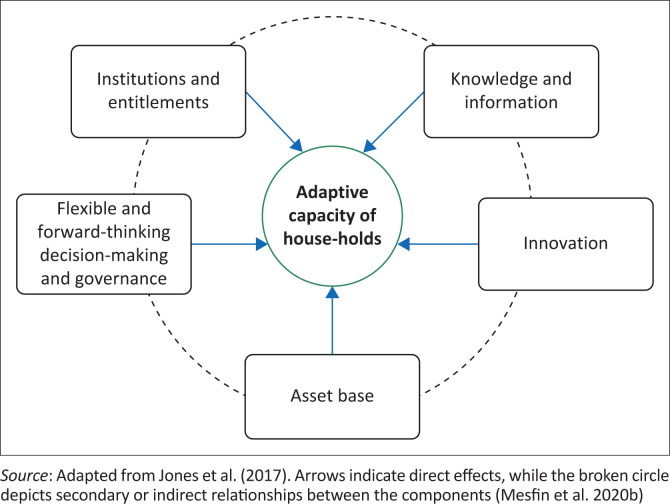
The local adaptive capacity framework for assessing the adaptive capacity of households.

Figure 1 lists the asset base, institutions and entitlements, knowledge and information, innovation and flexibility. It leaves out the community’s underlying causes of poverty, such as the number of economic resources and the efficiency and adaptability of local institutions. Despite this framework’s strength, gender dynamics at the household level and biophysical components – which are crucial for comprehending smallholder farmers’ ability to adjust at local levels – remain implicitly unacknowledged. Moreover, the framework provides a means of examining (and searching for) characteristics that typically support adaptive capability; it does not define the characteristics of an adaptable system.

### The adaptive capacity wheel

**FIGURE 2:**
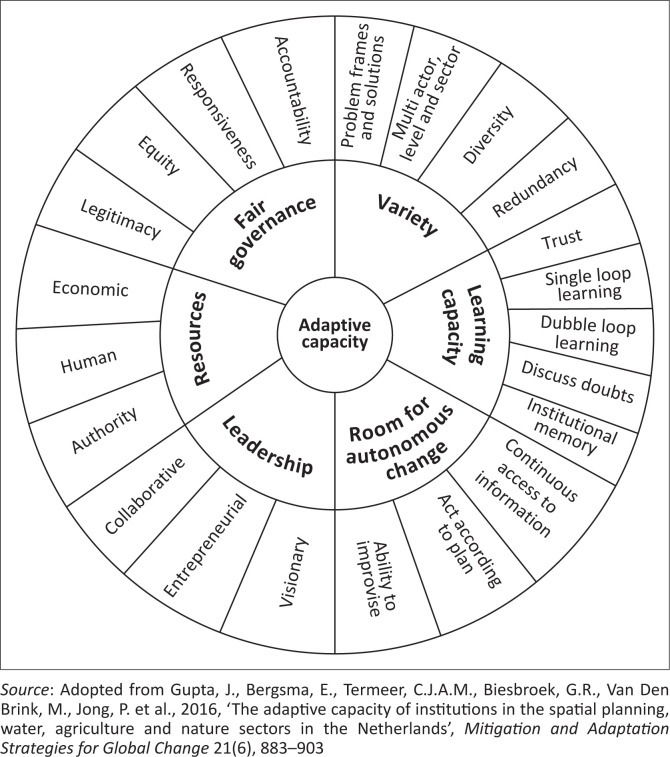
The adaptive capacity wheel.

The purpose of this framework’s development was to evaluate an institution’s capacity to adapt to support the adaptative capacity of society and to be modified by society (Gupta et al. 2016). Figure 2 provides a more detailed breakdown of the six institutional features: leadership, resources, diversity, fair governance, opportunity for autonomous change and learning capacity.

This wheel assists researchers and social actors in determining whether institutions support society’s ability to adapt to climate change and in concentrating on institutional redesign needs.

However, this only addresses one aspect of the variables that influence adaptive capacity; it ignores other factors mentioned in SLF that are crucial for determining the adaptive capacity of smallholder farmers household level, such as biophysical variables that influence household-level individual adaptability.

Consequently, its use would not provide a thorough grasp of the various degrees of adaptive capacity at the local level.

### Integrated adaptive capacity framework

According to Sechindra et al. (2022), social scientists describe adaptive capacity in terms of the biophysical and socioeconomic factors that support people’s potential to foresee, adapt to and recover from the effects of environmental change on both an individual and collective basis. People are impacted by environmental change on an individual and community level. The most obvious effects are frequently seen in losses that are personal in nature, such as the loss of life, livelihood, money, mental health and physical health (Steffen et al 2018). Because there is no clear link between the studies of persons and households and the effects of the environmental (biophysical) change processes, a large portion of the adaptive capacity literature in the environmental social sciences has concentrated on these topics (Siders 2019).

This is problematic because many of today’s most serious environmental changes – droughts that are more severe and last longer, floods that occur more frequently and the loss of vegetation, among others – have different effects on people’s ability to adapt over time and space than they do on communities (Haider et al 2018). Smallholder farmers need certain capacities to adapt to these kinds of environmental changes, and these capacities are often a product of societal and individual traits as well as nested capacities within groups. Therefore, it is crucial to evaluate smallholder farmers’ adaptive capacities at the local level, considering not only socioeconomic factors such as their capital assets base, social capital, gender dynamics, decision-making and governance, institutions and entitlements, knowledge and information but also biophysical factors such as the frequency of floods and droughts, as well as soil fertility among others.

When adaptation is approached holistically, it provides a better understanding of the various levels of adaptive capability than when it is conceptualised as a reaction to biophysical or socioeconomic causes alone.

To evaluate smallholder farmers’ ability to adapt to climate variability, this article examined the advantages and disadvantages of the various adaptive capacity frameworks. The result is a modified integrated adaptive capacity framework that includes social economic and biophysical components.

**FIGURE 3:**
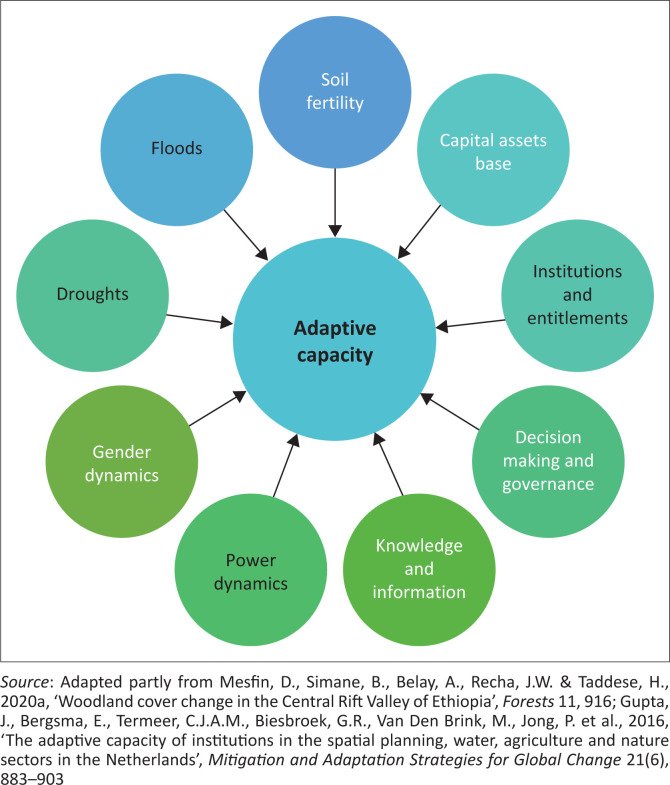
Researcher-modified integrated adaptive capacity framework.

The characteristics considered include capital assets base, institutions and entitlements, natural hazards, soil fertility, gender dynamics, power dynamics, flexible and forward-looking decision making, knowledge and information (Figure 3). The framework describes what an adaptive system looks like. It not only looks at features that tend to support adaptive capacity but also considers other aspects.

To comprehend how and why the adaptation capacity of smallholder farmers varies among various farming systems, socioeconomic situations and biophysical circumstances, more research is required. Furthermore, it is possible that the adaptive capacity can be depleted after an event before the next one happens in hazard-exposed places such as Malawi and Karonga (Botha, Nkoka & Mwumvaneza 2018) where disasters are severe and occur more frequently. The danger of adverse effects would rise because of this degrading capability considering climate variability, and this risk would increase when combined with an increase in the frequency of hazards. Because of this, this study is essential to understanding the nature and factors that influence small-holder farmers’ adaptation capacity considering their socioeconomic backgrounds, biophysical factors and methods of acclimatisation. The policies for allocating sufficient resources to guarantee smallholder farmers’ capacity to adapt to unforeseen climate threats can be further informed by these results. It is possible to identify villages that are particularly vulnerable to climate variability and determine investments for adaptation to future impacts of climate variability in Malawi and the study area by knowing the level of adaptive capacity that smallholder farmers have in each village to adapt to climate variability.

## Research methods and design

### Description of the study area

**FIGURE 4:**
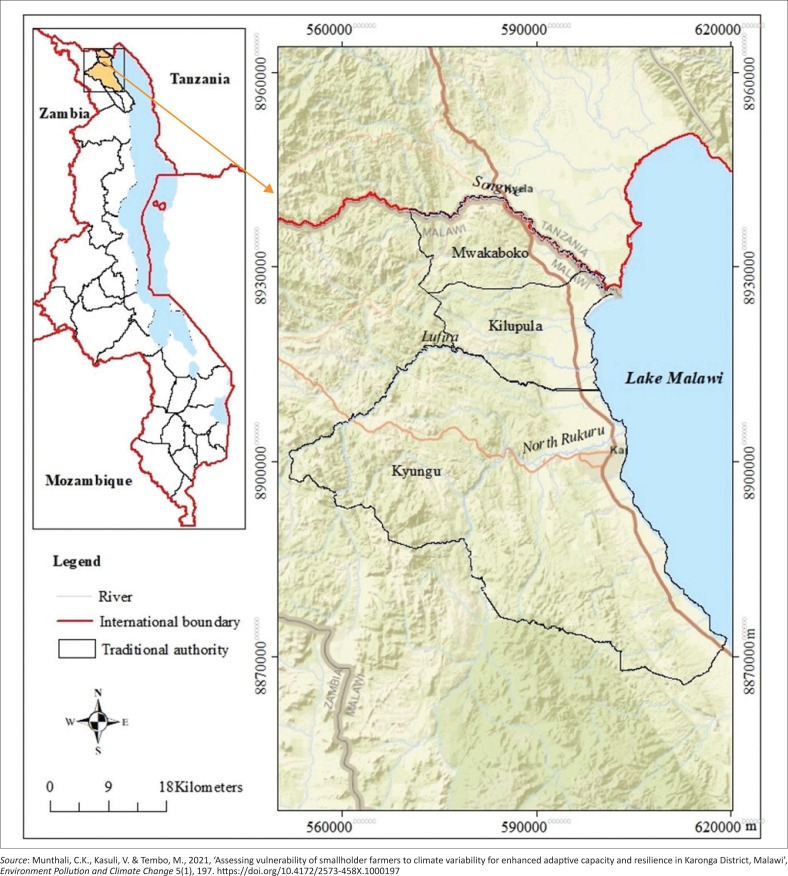
Location of Karonga within Malawi.

This study was conducted in Karonga district, which is in the Northern region of Malawi. It is bordered by Chitipa District in the West, Rumphi District in the South and Tanzania in the North (Munthali, Kasuli & Tembo 2021) (see Figure 4).

#### National context

Malawi’s continued reliance on rainfed, subsistence agriculture restricts its ability to grow, makes it more vulnerable to weather shocks and leads to food insecurity (World Bank 2022). With an 18 million pollution level, rain-fed agriculture accounts for a large portion of Malawi’s economy in terms of jobs, foreign exchange and means of subsistence (NSO 2018). Malawi’s reliance on agriculture to fuel economic growth has not helped many people escape poverty; however, between 2010 and 2019, the percentage of households earning their primary income from agriculture fell from 70% to less than 50%. Only 29% of people in 2019 derived their income from agriculture, down from 56% in 2010 (World Bank 2022).

As a result, Malawi’s economy is susceptible to both severe droughts and flooding. Climate change, population growth and environmental degradation are the main causes of the rising intensity and frequency of disasters. (Government of Malawi 2018). Malawi is more vulnerable to climate change because of several issues, such as rapid population expansion, reliance on rain-fed agriculture, high rates of malnutrition and insufficient electricity supplies. Extreme weather and climate conditions, such as more frequent and severe droughts and floods combined with rising temperatures, have a detrimental effect on the productivity of fisheries, forests and agriculture – all of which supply vulnerable populations with fuel, food, income and other environmental services.

Key resilience indicators (poverty, food insecurity and malnutrition) indicate that many households (HHs) in Malawi are still caught in a cycle of vulnerability even though some development indicators have improved in recent years (GoM 2017b, 2017d). These are not equipped to handle shocks and stressors, absorb them or change with the environment (GoM 2015a, 2015b, 2015c). The majority of Malawi’s rural population (84% – GoM 2022b) makes rain-fed small-scale agriculture their main source of income.

#### Karonga District context

**FIGURE 5:**
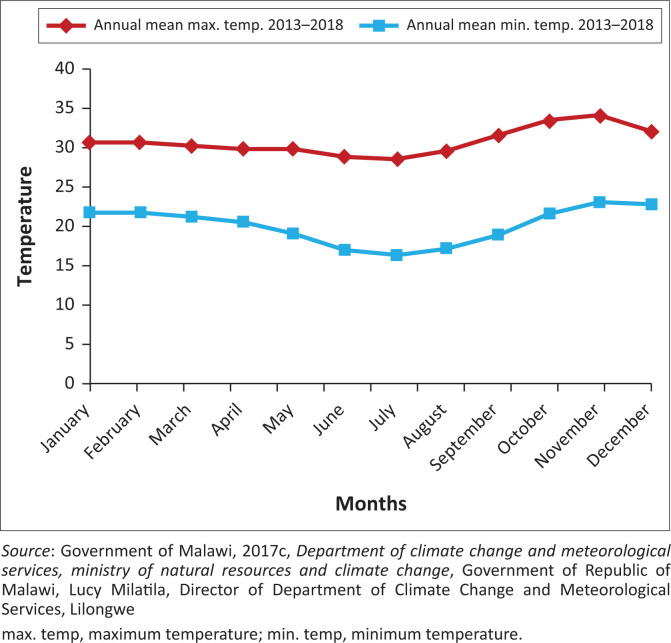
Mean maximum and minimum temperature from 2013–2018.

With two distinct seasons, rainy (November to May) and dry (June to October), Karonga District, has a subtropical climate. All year round, the average temperature is 24°C. June and July have lower temperatures, while October and November are the hottest months. The district has north-easterly winds in October and November and south-easterly winds from April to September (GoM 2022a). The district has pleasant weather, with an average yearly temperature of 24°C. Between June and July, mean low temperatures fall between 17°C and 23°C. From October through November, the average maximum temperature is between 29°C and 34°C (Figure 5).

**FIGURE 6:**
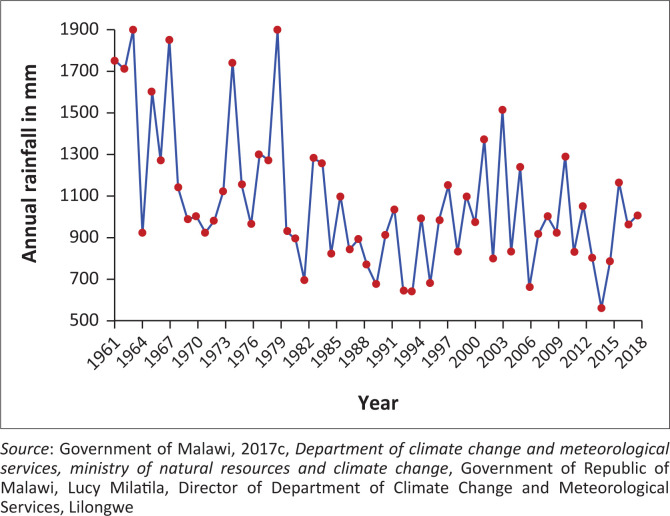
Annual rainfall from 1961–2019.

The mean annual rainfall for the district is about 1400 mm. The highest rainfall in the district is experienced around Mwangulukulu (Traditional Authority Mwakaboko) area where normally annual rainfall amount of around 3000 mm is experienced because of the topography. Most of the rains in the district fall between March and April when the maintain-bearing system, the Inter Tropical Convergence Zone (ITCZ), is anchored within the district as it progresses northwards. This triggers high intense rainfall that results in floods in the low-lying areas of the district. However, the Arabian ridge from the east stretches into the district and dry spells or droughts in extreme cases are induced over Karonga District (see Figure 6) (GoM 2022b; Munthali et al. 2021).

The socioeconomic progress the district has made over the previous 15 years is being undermined by natural hazards. Karonga’s narrow economic basis, small agro-processing companies, excessive reliance on rain-fed agriculture and usage of biomass for domestic energy all contribute to the worsening of the situation. Increased rural poverty, population pressure on finite land resources, land degradation from agricultural expansion and the cultivation of marginal lands and increased deforestation to meet rising energy, food and construction demands all contribute to the worsening of the situation. As it works to ensure sustainable livelihoods for its people, Karonga District Council is deeply concerned about the loss of human, natural, financial, social and physical capital brought on by the negative effects of climate change, particularly floods and droughts (GoM 2022c).

Furthermore, within Malawi’s institutional structure and agricultural governance, Karonga district is acknowledged as part of the Karonga Rural Development Programme (RDP). Like all of Malawi’s districts, Karonga RDP was chosen because it is putting into practice a pluralistic, demand-driven policy, with several non-governmental organisations working in tandem with the government to help small holder farmers become more resilient to climate change and agricultural extension.

According to Table 1, the district’s total estimated arable land is 67 100 hectares, of which 60 702 hectares are planted with crops. The least amount of land is farmed in the Kaporo North Extension Planning Area, while the most are in Mpata EPA. The district’s average landholding size is 0.75 hectares.

**TABLE 1:** Selected Extension Planning Areas (EPAs) for the study.

TA	EPA	Amount of rainfall (mm)/year				
-	-	2013/14	2014/15	2015/16	2016/17	2017/18
Mwakaboko	Kaporo N	17951.5	1367.9	2122.8	1636.9	1645.8
Kilupula	Kaporo S	1567.4	801.2	1194.1	1398.5	2007.6
Kyungu	Mpata	890.4	993.2	1591.2	1111.8	1765.7

*Source*: Government of Malawi, 2018, *District Meteorological Office, Ministry of Natural Resources and Climate Change*, Government of the Republic of Malawi, Lilongwe

TA, traditional authority.

More than 80% of people living in the RDP rely heavily on agriculture for their income and means of subsistence. The main crops grown there are maize, cassava, rice, cotton, groundnuts, sweet potatoes and pigeon peas, grown by more than 5% of farmers, and minor crops such as beans, millet, sesame, tobacco, sorghum, soya, sunflower and ground beans are grown by less than 5% of farming families (GoM 2022a). According to Karonga SEP (2022), each farming household in the district has an average land holding size of 0.55 hectares (GoM 2022a).

The District Agriculture Office is headed by the Director of Agriculture, Environment and Natural Resources (DAENAR), EPAs are led by the Agriculture Extension Development Coordinators (AEDC) and Sections are staffed by AEDOs.

#### Selected Extension Planning Areas (EPAs) context

The case studies for this research were Mpata EPA, Kaporo South and Kaporo North EPAs. These EPAs were selected because they have a smaller number of extension officers per extension planning area in the district with an average of 2318 extension farmer ratio, which is like Zambia where extension farmer ratio stands at 1 to 1:1200 for crops production and up to 1:3000 in the case of livestock production (Livune 2022).

The standard extension farmer ratio is 1:750; however, none of the extension planning areas meets this standard. It is an indication that a good number of farmers are not reached with extension and advisory services. Maize, rice, cassava, cotton and sweet potatoes are the major crops in the selected EPAs for the study. The rainfall patterns in these EPAs have been changing and unpredictable over the years because of climate change effects. Floods tend to occur more in the three EPAs of the district resulting in destruction of shelter, loss of human life, livestock and loss of crops. It also affects social and economic services. Because of climate change and global warming, the three selected EPAs have been experiencing incidences of drought over the past 5 years.

The population in the three EPAs obtain livelihood from the diverse resources in the form of food (fish, game, fruits and honey production), construction materials, fuelwood, pharmaceuticals, etc. Smallholder farmers in the EPAs practice various technologies to control soil erosion and conserve water. These technologies include swales, soak pits, infiltration basins, integrated soil fertility improvement, conservation agriculture and irrigation farming. They also use marker ridges, ridge realignment, box ridging, gully reclamation to reduce runoff and soil erosion, conserve soil moisture for plant growth, prevent siltation and flooding and increase groundwater supply. Implementation of these technologies has helped them to attain a decreasing rate of soil erosion to around an average of 9.4 tons per hectare per year and a maximum of 20.5 tons per hectare per year, which is lower than the national soil loss rate of 29 tons per ha per year. Table 2 depicts the farming population and farming practices in the three EPAs (District Agricultural Development Office).

**TABLE 2:** Farming population and farming practices in the three Extension Planning Areas (EPAs).

Variable	Mpata	Kaporo South	Kaporo North
Farming households	19 574.00	13 635.00	12 545.00
Average rainfall (mm)	1765.70	2007.60	1645.80
Extension farmer ration	2174.00	2273.00	2509.00
Yield (Kg^-ha^)	1893 (maize)	2507 (rice)	2507 (rice)
Arable land (ha)	14 680.00	10 226.00	9408.00
Average land holding size	0.74	0.74	0.75
Potential irrigable land area (ha)	64.00	100.00	25.00
Smallholder irrigated area (ha)	8.60	29.60	10.00

*Source*: Government of Malawi, 2017a, *Director of Agriculture, Environment and Natural Resources (DAENAR) Office Annual Extension Planning Area data*, Rita Khunga, Government of Republic of Malawi, Lilongwe

### Study design

This research covered a wide range of issues from socioeconomic to biophysical aspects and needed both quantitative and qualitative data. We adopted a convergent parallel mixed-methods design, where both quantitative and qualitative data are collected at about the same time and converged to provide a comprehensive analysis of the problem (Mesfin et al. 2020b).

This study employed a mixed-methods research design, which included the integration of quantitative and qualitative data collection methods and analysis.

### Sampling framework

The survey was conducted using a multiphase sampling technique. The choice of the study location was the initial step. The study site was chosen specifically because it is among Malawi’s most susceptible regions to climatic fluctuation and change (GoM 2022). There were additional factors considered in the selection process, such as the ecosystem’s sensitivity to environmental changes and its history of deforestation (Manda 2017). The research area’s group village headmen, the smallest governmental administrative units, were chosen in the second stage.

To gather the necessary data for this study, three samplings were carried out: one of smallholder farmers, to whom questionnaires were given and administered; another of smallholder farmers in the focussed group discussion, to whom a checklist of questions was given to seek consensus on certain issues (weighting of adaptive capacity indicators). A multi-stage sampling procedure was employed in the study to select smallholder farmers to serve as study participants. The district’s three traditional authorities (TAs), Kyungu, Mwakaboko and Kilupula, were purposefully chosen in the first stage out of five TAs as the district experiences frequent floods and droughts because of climate change and variability. Six group village headmen (villages) were chosen at random from the six TAs in the second stage. The study employed proportionate and simple random selection techniques to pick smallholder farmers as responders, considering the number of smallholder farmers in each village, as determined by the village listing. Adams (2020), adapted from Israel (1992), used the probability proportional to size (PPS) approach to determine the sample size from the selected village. This helped to ensure equal representation of households in each village. As a result, Slovin’s sample size calculation algorithm was employed to determine the final sample size in the following equation (Equation 1):


$$n=\frac{N}{\left(1+Ne2\right)}$$ [Eqn 1]

where:

*n* is the sample size,

*N* is the population, and

*e* is the acceptable margin of error.

Based on the 2645 agricultural households in the six villages (Karonga District Agriculture Office) as the total population, the mathematical calculation yielded 345 with a margin of error of 5%. Each of the six communities received a proportionate share of the sample.

In order to do this, the list of all farm household heads was taken in order to obtain sampling frames for each village. A predefined sample size of each village was divided by the total number of households to arrive at a specific sampling interval, ‘K’.

Next, using the lottery approach, a number between 1 and the sampling interval ‘K’ was chosen. This number, known as the random start, was utilised as the first number in the sample. After that, each village’s necessary sample size was reached by selecting the head of every Kth household. Feige and Marr (2012) state that systematic sample units are uniformly distributed over the population, indicating that the population is conceptually homogeneous within the various villages, which is why systematic sampling was used. The houses of smallholder farmers, evenly dispersed throughout the corresponding villages, serve as the sampling units in this instance.

According to the formula, the sample size for the four villages is 345. Table 3 is a summary of the population and derived sample sizes (Munthali et al. 2021).

**TABLE 3:** Designated sample sizes for the different strata.

No	Traditional authority (TA)	Group village head	Total population of farming families	Total sample size
1	Kyungu	Mwahimba	679	89
2		Zindi	364	48
3	Kilupula	Mwenitete	234	30
4		Mwaulambo	148	20
5	Mwakaboko	Mwakaboko	563	73
6		Mwangulukulu	657	85

*Source*: Adapted from Munthali, C.K., Kasuli, V. & Tembo, M., 2021, ‘Assessing vulnerability of smallholder farmers to climate variability for enhanced adaptive capacity and resilience in Karonga District, Malawi’, *Environment Pollution and Climate Change* 5(1), 197. https://doi.org/10.4172/2573-458X.1000197

### Data collection

Data were collected from both secondary and primary sources for the chosen villages of Karonga District, which was utilised to assess the adaptive capacity of smallholder farmers. An integrated indicator-based framework of determinants and related indicators was used to assess the adaptive capacity of smallholder farmers, and it was based on a comprehensive evaluation of scientific literature. A systematic questionnaire was created based on the indicators chosen from the literature reviews to apply the integrated indicator-based framework in the study areas. The smallholder farmers were subsequently given the survey questionnaire. At the time of the study, these communities were not being piloted for any adaptation to climate change projects.

This sampling criterion ensures that data acquired from the field are relatively free from the impacts of capacity development projects. A questionnaire was administered through face-to-face interviews. It had been developed in such a way that it gathered information on the different components of adaptive capacity guided by the integrated indicator-based framework (Mesfin et al. 2020b).

A pre-test of the questions and specific parts of the questionnaire was conducted on the smallholder farmers in the study area. The survey questions were prepared in English and then translated into the local language (Kyangonde) to guide the data collectors during interviews. A pre-test was necessary to assess whether the instruments were appropriate and suited to the study. Necessary amendments were made by deleting and modifying confusing and sensitive questions based on the comments from experts and observations of households’ responses (Munthali et al. 2021).

Following the principle of ‘informed consent’, the survey participants were informed about the anonymised use of the survey results and the protection of privacy. For the expert interviews, the names of the organisations of the expert interviewees were anonymised to protect the respondent’s confidentiality.

#### Household surveys

A household survey was conducted to collect information on the adaptive capacity of smallholder farmers who were the key focus of the study on climate change and variability. In particular, the survey collected information on the adaptive capacity of smallholder farmers’ households to climate change and variability in demographic and economic household characteristics, livestock and crops production, gender and power dynamics, institutional support, capital assets, access to extension services, credit access, hazards and disaster occurrence, livelihood diversification, institutional support, knowledge and information, family size, farmland location, soil erosion rate, land fertility level, land exposure to flood, crop productivity on temporal scale, distance to agricultural input markets and input utilisation, just to highlight some. Data were collected by agriculture extension workers who were employed as enumerators.

#### Focus group discussions

To cross-check and validate responses from household respondents, a total of six focus group discussions (FGDs) were held separately from the sampling villages, with a gender parity of five men and five women for each FGD. Ten people made up each focussed group discussion: two community leaders, seven smallholder farmers from each village and a Ministry of Agriculture representative. For the three-member research team, one asked questions, the other recorded responses and taking pictures was the responsibility of the third member. The purpose of this focussed group discussion was to assess and score indicators of adaptive capacity to climate change-based community viewpoint.

#### Field observations

Direct field observations were conducted to validate data gathered through household surveys. Vulnerable areas were documented through photographs by using a digital camera. Field observations focussed on bio-physical characteristics, land degradation, flood-affected areas, water resources and vegetation cover and land management practices.

### Data analysis

The five livelihood assets, that is natural, physical, financial, human and social assets, form an integral part of adaptive capacity in the existing literature (UNDP 2020). Mesfin (2020a) has challenged assessments that just rely on livelihood assets for neglecting to include crucial contextual information and the underlying institutional and social mechanisms that generate capacity. In addition to the livelihood assets, this study incorporated additional dimensions: decision-making, gender dynamics, power dynamics, knowledge and information, institutions, governance and frequency of floods, among others as depicted in the integrated conceptual framework (Figure 3). Relevant indicators were included to assess each dimension and provide a more complete picture of adaptive capacity at the local level. A detailed description of how each of the components was interpreted against the framework and the relevance of each indicator in enhancing the adaptive capacity of households in the face of a changing climate is provided in this section.

#### The asset base

The asset base is considered one of the five major dimensions of adaptive capacity. Availability and access to these assets will allow the system to respond to evolving circumstances. The indicators used to measure each asset type and their descriptions are presented here.

#### Natural assets

Grazing land, water resources and farmland (soil fertility) are examples of natural assets. Because farmland productivity indicates agricultural productivity for smallholder farmers, it is utilised as a measure of farmland assets rather than just size. An important factor in high agricultural productivity is soil fertility. Given that the study areas’ economies are focussed on agriculture, having rich soils and consistent rainfall would increase agricultural productivity and the ability of smallholder farmers to adapt. In addition, households with a greater proportion of productive farmland will have more be less vulnerable to climate-related dangers than households with a higher proportion of less productive farmland.

Water is a critical resource in the study area both for domestic consumption and agriculture. Hence, access to water resources and the quality of water for household use are assessed as one of the important natural assets aligned with the adaptive capacity of smallholder farmers.

#### Physical assets

Indicators for the physical assets include the type of house, ownership of mobile phone and radio, access to electricity and input and output markets. Possession of a better-quality house, besides being used as an indicator of socioeconomic status, will improve the adaptive capacity of smallholder farmers to endure the risks from extreme weather and other natural hazards. Ownership of mobile phones and radios increases adaptive capacity by creating access to the market, weather and climate-related information like early warning systems. Better information will enable households to make informed decisions, particularly on their farming activities and to take proactive adaptation measures against climate-induced disasters. The availability of the output and input markets with many other services such as extension service is linked to the adaptive capacity of smallholder farmers as they would find it easy to sell livestock and crops and would most often not compromise the price because of the distance factor other than farm gate prices set by Malawi government. They also have less chance of generating income from alternative sources such as non-farm labour, which is important in securing livelihood, particularly during periods of crop failure or floods.

#### Financial assets

Indicators of financial assets include gross annual income, livelihood diversification, access to credits and ownership of livestock. Gross annual income is derived from both cash and non-cash income sources. Access to credit facilities can be used to make productive investments that in turn are important to build their adaptive capacity in the long run and use it as a buffer during times of need.

#### Human assets

The highest educational qualification in the household, the number of training events attended by household members, thereby reducing their capacity to adapt to the impacts of climate change.

#### Social assets

Households can make some profitable investments or deal with seasonal food shortages by having access to financing through social ties. The credit may be given in cash, in kind or in both. Typically, livestock, forest products or agricultural items are sold to repay the debt. It is crucial in rural areas where access to official credit and savings institutions is limited. Therefore, improved credit availability suggests improved adaptability.

#### Institutions and entitlements

The existence of an appropriate and evolving institutional environment that ensures fair and equitable access, as well as entitlement to key resources and assets, is a fundamental feature of adaptive capacity. Gender inequality among smallholder farmers lowers the adaptive capacity of the powerless especially women smallholder farmers in the study area.

#### Knowledge and information

If smallholder farmers have access to sufficient knowledge and information on climate change and variability, they are better equipped to adapt to climate-related changes. Therefore, a key component of adaptive capacity is a system’s ability to gather, evaluate and share knowledge and information in support of adaptation efforts. Additionally, this enhances smallholder farmers’ perceptions of climate unpredictability, which is crucial for their adoption of climate-smart agricultural technologies and increases their capacity for adaptation.

#### Gender dynamics and power dynamics

Gender-climate change discussion has received scholarly attention over the past three decades. It begins with the widely held belief that gender is not a factor in climate change (Corcoran-Nantes & Roy 2018). The pool of capacities that people must access, control and manage the skills and resources needed to develop adaptive capacities is, in part, determined by the gender norms of their community (Phan, Jou & Lin 2019). The Intergovernmental Panel on Climate Change (IPCC) has broadened the scope of its discussion of adaptive capacity to human migration, cultural losses, institutional leadership, human rights and socio-ecological systems in its sixth assessment report (AR6) (IPCC 2022).

Gender equality is directly linked to the adaptive capacity of smallholder farmers. In the study area, 70% of agricultural production is performed by women, and as such if they are not involved in decision-making, control of resources would lend them with low adaptive capacity.

#### Frequency of floods

The assessment of adaptive capacity requirements varies depending on the threat being handled, scale, demographics and physiography. The ability of smallholder farmers to adapt would be destroyed by the frequency and severity of floods. The IPCC 2022 report states that there is an inverse relationship between system sensitivity to climate variability and adaptive capacity.

#### Measuring adaptive capacity

In this study, to determine the various household indicators of adaptive capacity, the principal component analysis (PCA) was applied. The PCA helped in reducing the dimensionality of the input variables and identifying significant indicators that were included in the model (Mbaziira et al. 2023). For various indicators identified during the survey, the PCA established groups of homogeneous and heterogeneous indicators within household dimensions.

### Principal component analysis

A group of potentially correlated variables (with a similar property, such as locations in space or time) can be transformed into a set of uncorrelated variables that reflect the variability in the underlying data using PCA, an ordination-based statistical data exploration technique. Because PCA is a non-parametric analysis and is independent of any premise regarding the probability distribution of the data, it can be used to reveal patterns within multivariable data (Mesfin 2020a). The eigenvalues connected to the vector for each principal component (PC) were used to rank the PCs according to their significance (i.e. the amount of variability in the data they capture).

For this article, the loadings from the first component of PCA were used as the weights for the variables and the weighted value for each variable varies between −1 and +1. The sign (+ or −) of the variables denotes the direction of the relationship with other variables used to construct the respective index.

#### How principal component analysis was conducted

Principal component analysis was used to generate composite indices by using the ‘Eigenvalue-greater-than-1’ rule proposed by Kaiser. After retaining all the components with eigenvalues greater than 1, factor analysis in SPSS generated factor loadings for all indicators, which were used as weights. Principal component analysis is a multi-stage kind of analysis. Table 4 shows a summary of how the analysis was conducted to finally arrive at the indices.

**TABLE 4:** Biophysical indicators and socioeconomic indicators used as proxies for principal component analysis.

Biophysical indicators (physical and natural capital)	Socioeconomic indicators (capital, asset, social, knowledge and information, gender and power dynamics)
X1: Share of more productive land possessed	X1: Ownership of livestock
X2: Access to water resources	X2: Livelihood Diversification
X3: Share of less productive land possessed	X3: Gross annual income
X4: Irrigated land	X4: Exclusion from food for work
X5: Soil erosion challenge faced	X5: Support from the community
X6: Agricultural tools	X6: Access to credit through social contacts
X7: Have a mobile phone	X7: Age of household head
X8: Walking distance to the nearest road	X8: Highest qualification in the household
X9: Type of house	X9: Knowledge and information on adaptation strategies
X10: Flooding disaster frequency	X10: Adequacy of knowledge and information
*X11: Drought disaster frequency*	X11: Access to phone or radio
X12: Availability of a dyke	X12: Local institutions relied upon for support during times of climate hazard
X13: Loss of livestock because of floods	X13: Dependence on outside support from local institutions
X14: Access to inorganic fertiliser	X14: Equitability of access to the support provided by local institutions
X15: Availability of organic fertiliser	X15: Flexibility in decision making
X16: Availability of camping sites during disasters	X16: Capacity to deal with hazards
X17: Affected by deforestation	X17: Who makes decisions at the household level
X18: Ownership of woodland	X18: Gender of the household head
X19: Dependance on charcoal burning for livelihood	-
X20: Total crop yield	-

The required underlying assumptions for PCA were fully met. To check the robustness of the model, two statistical tests, the Kaiser–Meyer–Olkin (KMO) test of sampling adequacy and Bartlett’s test of sphericity were used. Components that increased vulnerability were considered positive, and those that reduced vulnerability were viewed as negative (Mbaziira et al. 2023).

Varimax rotation was used to simplify the structure of the underlying dimensions and produce more independence among the factors. The varimax rotation also minimised the number of variables that loaded high on a single factor, thereby increasing the percentage variation between each factor (Armaș & Gavriș 2013). The Kaiser criterion (Eigenvalues > 1) was applied for the component selection.

This stepwise exclusion approach was carried out and iterated until the variables and components were stable and statistically robust (Mavhura, Manyena & Collins 2017). To avoid the undue influence of any single variable on the principal components, the variables were standardised by using z-scores. Afterwards, PCA was performed on z-standardised values, which was explained in data and transformation with acceptable KMO sampling adequacy (KMO > 0.6) and Bartlett’s test of sphericity values (*p* < 0.05); thus the sampling adequacy was satisfied (Mavhura et al. 2017). The subset of the indicators was selected based on factor loadings and the correlation matrix (Table 4). The indicators with higher factor loadings were preferred. The first case was when the categorical variables in the mixed dataset were dummy variables, the usual PCA was fitted.

Using literature coupled with an understanding of the study area, 38 indicator variables were identified, which were used as proxies for the PCA. Twenty of these were for biophysical adaptive indicators and 18 were for socioeconomic adaptive indicators. Table 4 is a summary of the variables.

### Ethical considerations

Ethical clearance to conduct this study was obtained from the National Commission for Science & Technology, Health, Social Sciences and Humanities Division (reference no.: NCST/RTT/2/6). In addition, the researcher ensured that participants were fully informed and gave their informed consent. Participation in the study was voluntary, and participants had the freedom to withdraw at any time. This means that research subjects were always aware of the study’s nature and goals and were required to provide consent before participating.

Participant safety was prioritised, ensuring that no risks or injuries were encountered by the human respondents. The confidentiality and anonymity of the participants were consistently maintained, with privacy being respected at all times. By assuring the participants that they would not be subjected to any deceptive or untrustworthy actions throughout the research process or in the dissemination of its findings, trust was established.

## Results

The Kaiser–Meyer–Olkin test must yield values greater than or equal to 0.6 (KMO ≥ 0.6) as a requirement for PCA. In this study, it indicates that the variables were suitable for PCA; this is because the KMO test for the biophysical analysis yielded KMO = 0.746, while for the socioeconomic indices, the value yielded was KMO = 0.606; thus, the sampling adequacy was satisfied. The Bartlet’s test of sphericity must be significant at (*p* < 0.05). In this study, for the biophysical indices, it was significant at *p* = 0.000, while for the socioeconomic analysis, it was significant at *p* = 0.000. Cutter et al. (2017) explain that taken together, both the KMO and Bartlett’s test of sphericity provide a minimum standard, which must be passed before conducting a PCA, and for this study, it was passed, as illustrated in Table 5 and Table 6.

**TABLE 5:** Kaiser–Meyer–Olkin and Bartlett’s test for socioeconomic factors’ principal component.

Measure of socioeconomic factor analysis applicability		Measured values
Kaiser-Meyer-Olkin Measure of Sampling Adequacy		0.606
Bartlett’s Test of Sphericity	Approx. Chi-Square	835.921
	*df*	153
	Sig.	0.000

Approx. approximate; *df*, degrees of freedom; Sig., significance.

**TABLE 6:** Kaiser–Meyer–Olkin and Bartlett’s test for biophysical factors’ principal component.

Measure of biophysical factor analysis applicability		Measured values
Kaiser-Meyer-Olkin Measure of Sampling Adequacy		0.746
Bartlett’s Test of Sphericity	Approx. Chi-Square	3152.763
	*df*	190
	Sig.	0.000

Approx. approximate; *df*, degrees of freedom; Sig., significance.

The extraction sums of squared loadings provide an overall picture of the amount of variance that each of the 38 chosen proxy indicators has been able to explain. However, we employ commonalities to pinpoint the precise percentage of each indicator’s variance that may be attributed to its seven primary components. The percentage of each variable’s variance that the major components can account for is referred to as the commonalities (Mavhura et al. 2017). A commonality must meet the following requirements to be recognised as proof that an indicator has been accurately represented by the extracted principal components: *h* < 0.5.

The commonalities for socioeconomic and biophysical aspects of adaptive ability are shown in Table 5 and Table 6, respectively.

Table 7 and Table 8 contain output from the rotated component matrix (RCM), sometimes also referred to as factor loadings. The RCM contains estimates of correlations between each of the variables and the estimated principal components. The greater the correlation, the better the component explains.

**TABLE 7:** Factor loadings showing the correlation between indicators and principal components for socioeconomic indicators.

Variables	Principle components						
	1	2	3	4	5	6	7
Ownership of livestock	0.790	-	-	-	-	-	-
Livelihood diversification	0.747	-	-	-	-	-	-
Gross annual income	−0.643	-	-	-	-	-	-
Exclusion from food for work	-	−0.831	-	-	-	-	-
Support from the community	-	0.771	-	-	-	-	-
Access to credit through social contacts	-	0.432	0.394	-	-	-	0.355
Age of household head	-	-	−0.732	-	-	-	-
Highest qualification in the household	-	-	0.707	-	-	-	-
Knowledge and information on adaptation strategies	-	-	-	0.674	-	-	-
Knowledge and information	-	-	-	−0.645	-	-	-
Access to phone or radio	-	-	-	0.595	-	-	-
Local institutions relied upon for support during climate hazard	-	-	-	-	−0.686	-	-
Dependence on outside support from local institutions	-	-	-	-	−0.569	0.453	-
Equitability of access to support from local institutions	-	-	-	0.319	0.543	-	-
Flexibility in decision-making	-	-	-	-	-	0.762	-
Capacity to deal with hazards	-	-	−0.373	-	-	0.640	-
Who makes decisions at the household level	-	-	-	-	-	-	0.733
Gender of the household head	-	-	-	-	0.483	-	−0.563

1, Financial; 2, Social; 3, Human Assets; 4, Knowledge and Information; 5, Institutions and Entitlements; 6, Flexible and Forward-looking Decision-making; 7, Gender and Power Dynamics.

**TABLE 8:** Factor loadings showing the correlation between indicators and principal components for biophysical indicators.

Variables	Principle components						
	1	2	3	4	5	6	7
I share of more productive land possessed	0.792	-	-	-	-	-	-
I have access to water resources	0.784	-	-	-	-	-	-
Share of less productive land possessed	−0.664	-	-	-	-	-	-
Irrigated land	0.647	-	-	-	-	-	-
Soil erosion challenge faced	−0.615	-	-	-	-	-	-
Agricultural tools	0.609	-	-	-	−0.378	-	-
Type of house	-	0.961	-	-	-	-	-
Availability of input and out-market	-	0.948	-	-	-	-	-
Have a mobile phone	-	0.935	-	-	-	-	-
Flooding disaster frequency	-	-	0.776	-	-	-	-
Availability of a dyke	0.344	-	−0.615	-	-	-	-
Loss of livestock because of floods	-	-	0.565	0.414	-	-	-
Access to inorganic fertiliser	-	-	-	0.806	-	-	-
Availability of organic fertiliser	-	-	-	0.738	-	-	-
Availability of camping sites during disasters	-	-	-	-	0.838	-	-
Affected by deforestation	-	-	0.450	-	−0.485	-	-
Ownership of woodland	-	-	-	−0.354	-	0.664	-
Dependance on charcoal burning for livelihood	-	-	-	-	0.374	0.510	-
Total crop yield	-	-	-	-	-	-	0.886

1, Natural Asset; 2, Physical asset; 3, Frequency of floods; 4, Fertility of farmland; 5, Infrastructure; 6, Vegetation loss; 7, Crop yield.

## Discussion

The main elements of adaptive capacity in this study and their respective contribution values are highlighted in Table 7 and Table 8. Each component or subcomponent is represented by a positive or negative weight, indicating a positive or negative relationship with other variables. Thus, the general value of the adaptability index was found, maintaining the transparency of the composite structure of this value based on the loading of the 14 main components. The relative share of subcomponents, or indicators, was presented using composite indices. The analysis found 14 key components, which included socioeconomic factors such as financial, social and human assets; knowledge and information; institutions and rights; flexible and forward-looking decision-making; gender and power dynamics and biophysical factors including natural resources, physical assets with flood frequency, crop fertility, infrastructure, vegetation loss and crop yield, were variables that affected both positively and negatively the ability of smallholder farmers to adapt to climate change.

Socioeconomic factors such as livestock ownership (0.790), community support (0.771), livelihood diversification (0.747) and decision-making by gender (0.733) showed the strongest positive influence on the adaptive capacity of smallholder farmers to climate change. In contrast, social capital – exclusion from food for work (−0.831), human capital – age of household (−0.732) and financial assets – annual gross income (−0.643) had a negative influence on the adaptive capacity of smallholder farmers to climate change (Figure 6). Because livestock is an expensive and considerable source of income, owning them is seen as a sign of wealth in the study area, where livestock ownership has been found to increase the adaptive capacity of smallholder farmers. Furthermore, there is a strong correlation between a household’s ability to adapt to a climate change disaster and its level of economic assets. This is because households with greater economic assets are more likely to be able to make changes to their way of life if they are exposed to hazards.

Adaptive capacity is enhanced by interpersonal relationships through networks and community levels. Communities should analyse current and past initiatives and adaptation processes through association and relationship building. Diversification of livelihoods in the household increases the adaptive capacity of smallholders because the limited availability of livelihoods increases the inability to avoid risks and increases the shocks and stress impact to which an individual or household is exposed.

The study found that decision-making by gender at the household level contributes positively to increased adaptive capacity. This is true when there are equal rights when it comes to decision-making at a household level between men and women. This is supported by the argument by Garcia, Tschakert and Karikari (2020) who state that women’s identity and role in societal platforms of dialogue and decision-making are defined by their relation to their husband or father’s position in that society.

Such social practices were found to be contributing to women’s lesser participation in community activities and lack of freedom to choose what they want to do, which might affect their adaptive capacity to climate variability (Rao et al. 2019). Men usually decide or have a vital say in women’s choice of activities and their social mobility, which eventually contributes to women’s restricted decision-making and bargaining power (Ferdous & Mallick 2019). Improving the understanding of gender relations would bring greater clarity to socio-structural power relations that might help improve women’s access to social elements that would enhance their adaptive capacity. Even though it will be challenging, changing social structures will need active engagement that recognises the challenges faced by women and gives them the opportunity to help with family and community adaptation to the impending effects of climate change.

Under biophysical factors, type of a house (0.961), distance to input and output market (0.948), having a mobile phone (0.945), access to inorganic fertiliser (0.806) and the share of more productive land possessed (0.792) had the highest influence on the adaptive capacity of smallholder farmers to climate variability in the study area. On the other hand, less productive land (−0.664) and deforestation (−0.485) had a negative influence on the adaptive capacity of smallholder farmers to climate change. The type of floor plan, foundation and roof materials were utilised as markers of the house’s quality to increase its ability to withstand flood hazards. Farmland’s contribution to the adaptive capacity was lessened when a larger portion of less productive land was owned, whereas ownership of more productive land had a greater influence on adaptive capacity. Similarly, rather than the quality of the water that was previously accessible, the scarcity of water resources for domestic and agricultural use had an impact on the score of water resources. The availability of quality portable water sources increases the adaptive capacity of smallholder farmers as it minimises exposure to water-borne diseases, which has a positive impact on the adaptive capacity.

The study reveals that while the commonly used asset base (socioeconomic factors) sounds more logical to influence adaptive capacity, it is multidimensional and determined by complex inter-relationships of many factors including biophysical factors at different scales, as reported by Mbaziira (2023). Thus, solely looking at the household’s asset base will not give a complete picture of smallholder farmers’ adaptive capacity to climate variability. It is therefore apparent that asset-based assessments need to be supplemented with the assessment of the intangible processes that play an integral part in determining the ability of a system to adapt to extreme weather and climate-induced hazards. The findings of this study demonstrate exactly that. The analysis also emphasises how crucial it is to evaluate adaptive capacity from a variety of angles. It offers a fresh viewpoint on adaptive capacity at the local level as well as an expanded area of analysis (Mozilla 2023). The five livelihood assets comprise the asset base, which is one of the five primary components utilised in this study to analyse determinants of adaptive capacity.

Based on the PCA results, development organisations and policymakers should concentrate on putting interventions into place that will increase the use of components that positively influence or contribute to smallholder farmers’ ability to adapt, as these components are the ones that do so more than other components.

However, this does not imply that the remaining components are not that important. It rather means that their contributions now are lower and that they should be improved by taking the right measures. Components such as exclusion from food for work, age of household head, gross annual income, less productive land and deforestation influence adaptive capacity negatively. The overall adaptive capacity index (ACI) was 0.48, meaning that most smallholder farmers in Karonga have limited adaptive capacity. This was determined by using the component loadings as the weights for the variables utilised in the ACI formulation. To transform them into valuable contributors, a great deal of work will need to be done. As a result, the report recommends taking steps to increase the use of resources that are not doing much to support smallholder farmers’ ability to adapt.

The integrated adaptive capacity framework reveals that a system as a whole functioning in an integrated manner has greater capability to improve adaptive capacity than either one or more components in isolation. It is, therefore, important to note that both socioeconomic and biophysical factors of a system form an integrated and systematic part of the adaptive capacity of smallholder farmers at the household level.

## Conclusion

Using the authors’ conceptualised integrated adaptive capacity framework, a household’s adaptable ability was analysed. This information (partially adapted from Mesfin 2020a and Gupta et al. 2016) confirmed that the asset base, knowledge base, knowledge and information, institutions and entitlements, flexible and forward-looking decision-making, gender and power dynamics, frequency of floods, fertility of farmland, infrastructure, loss of vegetation and crop yield facilitated by socio-ecological factors determined the adaptive capacity of smaller holder farmers in Karonga, Malawi.

The analysis showed that some component indicators contributed positively to adaptive capacity while others reduced it. Component indicators such as ownership of livestock, support from the community, livelihood diversification and gender of the decision maker, type of house (0.961), distance to input and output market, having a mobile phone, access to inorganic fertiliser and owning more productive land increased adaptive capacity of smallholder farmers, and component indicators such as owning a less productive land, deforestation, exclusion from food for work, age of household head and gross annual income reduced adaptive capacity of smallholder farmers to climate change.

The study’s conclusions revealed that while preserving component indicators that help smallholder farmers become more climate adaptive, more attention needs to be paid to improving the contribution of the component indicators that negatively affect their ability to adapt at the household level. Furthermore, by offering guidance on the relative contributions of the component indicators and factors indicating whether adaptive capacity is improving or decreasing, the study’s findings would help smallholder farmers improve their adaptive capacity.

This study is also thought to offer methodological elements for evaluating smallholder farmers’ adaptive capacity locally using an integrated adaptive capacity framework that considers biophysical factors that affect smallholder farmers’ adaptive capacity. Additionally, the selection of indicators through PCA methodologies aided in the creation of a comparative image of smallholder farmers’ ability to adapt to the threats associated with climate change and climate variability.

The study also showed that in addition to the assets’ availability, there are procedures and roles that must be in place for these resources to be mobilised. The composite index study findings give stakeholders – such as researchers, politicians and development actors – more information on which to base their decisions.

## References

[CIT0001] Adam, A., 2020, ‘Sample size determination in survey research’, *Journal of Scientific Research & Reports* 26(5), 90–97. 10.9734/jsrr/2020/v26i530263

[CIT0002] Amare, A. & Simane B., 2017, ‘Climate change induced vulnerability of smallholder farmers: Agroecology-based analysis in the Muger sub-basin of the upper Blue-Nile basin of Ethiopia’, *American Journal of Climate Change* 6(4), 668–693. 10.4236/ajcc.2017.64034

[CIT0003] Angeler, D., Fried-Petersen, H., Allen, C., Garmestani, A., Twidwell, D., Ching Chuang, W. et al., 2019, ‘Adaptive capacity in ecosystems’, *Advances in Ecological Research* 60, 1–24. 10.1016/bs.aecr.2019.02.001 31908359 PMC6944309

[CIT0004] Armaș, I. & Gavriș, A., 2013, ‘Social vulnerability assessment using spatial multi-criteria analysis (SEVI model) and the Social Vulnerability Index (SoVI model) – A case study for Bucharest, Romania’, *Natural Hazards and Earth System Sciences* 13(6), 1481–1499. 10.5194/nhess-13-1481-2013

[CIT0005] Asfaw, S., McCarthy, N., Lipper, L., Arslan, A., Cattaneo, A. & Kachulu, M., 2014, ‘Climate variability, adaptation strategies, and food security in Malawi’, ESA Working Paper No. 14–08, FA, Rome.

[CIT0006] Botha, B.N., Nkoka, F.S. & Mwumvaneza, V., 2018, ‘Hard hit by El Nino: Experiences, responses and options for Malawi’, Working Paper, World Bank Group, Washington, DC.

[CIT0007] Eastin, J., 2018, ‘Climate change and gender equality in developing states’, *World Development* 107, 289–305. 10.1016/j.worlddev.2018.02.021

[CIT0008] Engle, N.L., 2011, ‘Adaptive capacity and its assessment’, *Global Environmental Change* 21(2), 647–656. 10.1016/j.gloenvcha.2011.01.019

[CIT0009] Ferdous, J. & Mallick, D., 2019, ‘Norms, practices, and gendered vulnerabilities in the lower Teesta basin, Bangladesh’, *Environmental Development* 31, 88–96. 10.1016/j.envdev.2018.10.003

[CIT0010] Food and Agriculture Organisation of the United Nations (FAO), 2017, *Family farming knowledge platform*, FAO Publishers, Rome.

[CIT0011] Garcia, A., Tschakert, P. & Karikari, N.A., 2020, ‘“Less able”: How gendered subjectivities warp climate change adaptation in Ghana’s Central Region’, *Gender, Place & Culture* 27(11), 1602–1627. 10.1080/0966369X.2020.1786017

[CIT0012] Government of Malawi, 2013, *The state of Malawi climate in 2023*, Department of Climate Change and Meteorological Services, Government of Republic of Malawi, Lilongwe.

[CIT0013] Government of Malawi, 2015a, *Malawi’s National Adaptation Programmes of Action (Napa) under the United Nations Framework Convention on Climate Change (UNFCCC)*, 2nd edn., Director of Environmental Affairs, Ministry of Mines, Natural Resources and Environment Environmental Affairs Department, Lilongwe.

[CIT0014] Government of Malawi, 2015b, *Malawi national disaster risk management policy*, The Secretary and Commissioner for Disaster Management Affairs, Lilongwe.

[CIT0015] Government of Malawi, 2015c, *National Adaptation Programmes of Action (NAPA), under the United Nations Framework Convention on Climate Change (UNFCCC)*, 2nd edn., Tawonga Mbale-Luka (MS), Director of Environmental Affairs, Environmental Affairs Department, Lilongwe.

[CIT0016] Government of Malawi, 2016, *Malawi drought 2015–2016: Post-disaster needs assessment (PDNA)*, World Bank Group, Washington, DC.

[CIT0017] Government of Malawi, 2017a, *Karonga rural agriculture development, Director of Agriculture, Environment and Natural Resources (DAENAR) office annual extension planning area data*, Rita Khunga, Government of Republic of Malawi, Lilongwe.

[CIT0018] Government of Malawi, 2017b, *Malawi growth development strategy 2017–2022*, Ministry of Finance, Economic Planning and Development, Lilongwe.

[CIT0019] Government of Malawi, 2017c, *Department of climate change and meteorological services, ministry of natural resources and climate change. Weather and climate services and supporting forecasts and disaster response/management*, Lucy Milatila, Director of Department of Climate Change and Meteorological Services, Lilongwe.

[CIT0020] Government of Malawi, 2017d, *National Resilience Strategy*, Government of Republic of Malawi, Lilongwe.

[CIT0021] Government of Malawi, 2018, *District Meteorological Office, Ministry of Natural Resources and Climate Change*, Government of the Republic of Malawi, Lilongwe

[CIT0022] Government of Malawi, 2018a, *National Agricultural Investment Plan (NAIP). Prioritised and coordinated agricultural transformation plan for Malawi*, Gray Nyandule Phiri Secretary for Agriculture, Irrigation and Water Development Ministry of Agriculture, Irrigation and Water Development, Lilongwe.

[CIT0023] Government of Malawi, 2018b, *Malawi population and housing census main report*, Mercy Kanyuka, Commissioner of Statistics National Statistical Office (NSO), Zomba.

[CIT0024] Government of Malawi, 2018c, District meteorological office, *Ministry of natural resources and climate change*, Government of Republic of Malawi, Lilongwe.

[CIT0025] Government of Malawi, 2022a, *Karonga socio economic profile*, The District Commissioner, Karonga District Council, Karonga.

[CIT0026] Government of Malawi, 2022b, *Malawi vulnerability assessment report. Integrated food phase classification. IPC acute food insecurity analysis, June 2022 – March 2023*, Geresomo Victoria MVAC Chair, Lilongwe.

[CIT0027] Government of Malawi, 2022c, *Karonga socio economic profile*, Government of Republic of Malawi, Lilongwe.

[CIT0028] Gupta, J., Bergsma, E., Termeer, C.J.A.M., Biesbroek, G.R., Van Den Brink, M., Jong, P. et al., 2016, ‘The adaptive capacity of institutions in the spatial planning, water, agriculture and nature sectors in the Netherlands’, *Mitigation and Adaptation Strategies for Global Change* 21(6), 883–903. 10.1007/s11027-014-9630-z

[CIT0029] Haider, L.J., Boonstra, W.J., Peterson, G.D. & Schlüter, M., 2018, ‘Traps and sustainable development in rural areas: A review’, *World Development* 101, 311–321. 10.1016/j.worlddev.2017.05.038

[CIT0030] IPCC, 2022, ‘Annex II: Glossary [Möller, V., R. Van Diemen, J.B.R. Matthews, C. Méndez, S. Semenov, J.S. Fuglestvedt, A. Reisinger (eds.)]’, in H.-O. Pörtner, D.C. Roberts, M. Tignor, E.S. Poloczanska, K. Mintenbeck, A. Alegría, et al. (eds.), *Climate change 2022: Impacts, adaptation and vulnerability. contribution of Working Group II to the Sixth assessment report of the intergovernmental panel on climate change*, pp. 2897–2930, Cambridge University Press, Cambridge.

[CIT0031] Jones, L., Ludi, E., Jeans, H. & Barihaihi, M., 2017, ‘Revisiting the local adaptive capacity framework: Learning from the implementation of a research and programming framework in Africa’, *Climate and Development* 11(1), 3–13. 10.1080/17565529.2017.1374237

[CIT0032] Kapitza, S., Van Ha, P., Kompas, T., Golding, N., Cadenhead, N.C.R., Bal, P. et al., 2021, ‘Assessing biophysical and socio-economic impacts of climate change on regional avian biodiversity’, *Scientific Reports* 11, 3304. 33558621 10.1038/s41598-021-82474-z PMC7870812

[CIT0033] Livune, D., 2022, *An establishment of strategies that can make the agriculture extension education programme provided in Zambia effective: A case of Kazungula district*, dissertation for Master’s degree, Peace, Leadership and Conflict Resolution, Zimbabwe Open University, Harare.

[CIT0034] Manda, M. & Wanda, E., 2017, ‘Understanding the nature and scale of risks in Karonga, Malawi Environment & Urbanization Copyright © 2017’, *International Institute for Environment and Development (IIED)* 29(1), 15–32. 10.1177/0956247817692200

[CIT0035] Mataya, D.C., Vincent, K. & Dougill, A.J., 2020, ‘How can we effectively build capacity to adapt to climate change?’, *Insights from Malawi, Climate and Development* 12(9), 781. 10.1080/17565529.2019.1694480

[CIT0036] Mavhura, E., Manyena, B. & Collins, A.E., 2017, ‘An approach for measuring social vulnerability in context: The case of flood hazards in Muzarabani district, Zimbabwe’, *Geoforum* 86, 103–117. 10.1016/j.geoforum.2017.09.008

[CIT0037] Mbaziira, J., Anthony Egeru, B., Bamutaze, Y., Kisira, Y. & Nabiroko, M., 2023, ‘Assessing the dynamics of agropastoral farmers’ adaptive capacity to drought in Uganda’s cattle corridor’, *Climate Risk Management* 41, 100545. 10.1016/j.crm.2023.100545

[CIT0038] Mesfin, D., Simane, B., Belay, A., Recha, J.W. & Taddese, H., 2020a, ‘Woodland cover change in the Central Rift Valley of Ethiopia’, *Forests* 11, 916. 10.3390/f11090916

[CIT0039] Mesfin, D., Simane, B., Belay, A., Recha, J.W. & Schmiedel, U., 2020b, ‘Assessing the adaptive capacity of households to climate change in the Central Rift Valley of Ethiopia’, *Climate* 8(10), 106. 10.3390/cli8100106

[CIT0040] Montreux, C. & Barnett, J., 2017, ‘Adaptive capacity: Exploring the research frontier’, *Wiley Interdisciplinary Reviews: Climate Change* 8, e467. 10.1002/wcc.467

[CIT0041] Munthali, C.K., Kasuli, V. & Tembo, M., 2021, ‘Assessing vulnerability of smallholder farmers to climate variability for enhanced adaptive capacity and resilience in Karonga District, Malawi’, *Environment Pollution and Climate Change* 5(1), 197. 10.4172/2573-458X.1000197

[CIT0042] Niang, I., Ruppel, O.C., Abdrabo, M.A., Essel, A., Lennard, C., Padgham, J. et al., 2014, *Impacts, adaptation, and vulnerability. Part B: Regional aspects. Contribution of Working Group II to the fifth assessment report of the intergovernmental panel on climate change*, V.R. Barros, C.B. Field, D.J. Dokken, M.D. Mastrandrea, K.J. Mach, T.E. Bilir et al. (eds.), Cambridge University Press, Cambridge.

[CIT0043] Phan, L.T., Jou, S.C. & Lin, J.H., 2019, ‘Gender inequality and adaptive capacity: The role of social capital on the impacts of climate change in Vietnam’, *Sustainability* 11, 1257. 10.3390/su11051257

[CIT0044] Phiri, H., 2020, *Effect of land tenure on adoption of climate change adaptation: Evidence from Malawi*, Department of Agricultural and Applied Economics Lilongwe University of Agriculture and Natural Resources, Malawi.

[CIT0045] Rao, N., Mishra, A., Prakash, A., Singh, C., Qaisrani, A., Poonacha, P. et al., 2019, ‘A qualitative comparative analysis of women’s agency and adaptive capacity in climate change hotspots in Asia and Africa’, *Nature Climate Change* 9(12), 964–971. 10.1038/s41558-019-0638-y

[CIT0046] Sechindra, V., Smith, A., Chaffin, B., Nesbitt, H., Lohani, S., Gulab, S. et al., 2022, ‘Adaptive capacity beyond the household: A systematic review of empirical social-ecological research’, *Environmental Research Letters* 17, 063001. 10.1088/1748-9326/ac68fb

[CIT0047] Serrat, O., 2017, ‘The sustainable livelihoods approach’, in P. Ash, A.D. Atkinson, F.M. Alcaraz & H. Ear-Dupuy (eds.), *Knowledge solutions tools, methods, and approaches to drive organizational performance*, pp. 21–26, Asian Development Bank, Mandaluyong.

[CIT0048] Shikuku, K., Winowiecki, L., Twyman, J., Eitzinger, A., Perez, J.G., Mwongera, C. et al., 2017, ‘Smallholder farmers’ attitudes and determinants of adaptation to climate risks in East Africa’, *Climate Risk Management* 16, 234–245. 10.1016/j.crm.2017.03.001

[CIT0049] Siders, A., 2020, ‘Adaptive capacity to climate change: A synthesis of concepts, methods, and findings in a fragmented field’, *Wiley Interdisciplinary Reviews: Climate Change* 10, e573. 10.1002/wcc.573

[CIT0050] Singh, C., Deshpande, T. & Basu, R., 2017, ‘How do we assess vulnerability to climate change in India?’, *Environmental Change* 17, 527–538. 10.1007/s10113-016-1043-y

[CIT0051] Steffen, W., Rockstrom, J., Richardson, K., Lenton, T.M., Folke, C., Liverman, D. et al., 2018, ‘Trajectories of the Earth System in the Anthropocene’, *Proceedings of the National Academy of Sciences* 115(33), 8252–8259. 10.1073/pnas.1810141115 PMC6099852 30082409

[CIT0052] World Bank Group, 2022, *Malawi – Country climate and development report (English)*, World Bank Group, Washington, DC, viewed 29 May 2024, from http://documents.worldbank.org/curated/en/099545010272237260/P1772201ced75ce9182e7142761bde013662bca4fe42.

